# Antidepressant Effects of 20-Glucoginsenoside Rf in an L-AAA-Induced Mouse Model with Glial-Associated Molecular Alterations

**DOI:** 10.3390/cimb48090920

**Published:** 2026-09-09

**Authors:** Sangbeom Kim, Seung-Hun Cho

**Affiliations:** 1 Department of Clinical Korean Medicine, Graduate School, Kyung Hee University, Seoul 02447, Republic of Korea; pwn732732@gmail.com; 2 College of Korean Medicine, Kyung Hee University, Seoul 02447, Republic of Korea; 3 Department of Neuropsychiatry of Korean Medicine, Kyung Hee University Medical Center, Kyung Hee University, Seoul 02447, Republic of Korea; 4 Research Group of Neuroscience, East-West Medical Research Institute, WHO Collaborating Center, Kyung Hee University, Seoul 02447, Republic of Korea

**Keywords:** astrocyte, 20-glucoginsenoside Rf, L-alpha-aminoadipic acid, major depressive disorder, glial fibrillary acidic protein (GFAP)

## Abstract

Major depressive disorder (MDD) is a leading contributor to disability worldwide, yet current antidepressant treatments remain limited by incomplete remission, treatment resistance, and adverse effects. 20-glucoginsenoside Rf (20-gluco-Rf), a trace bioactive saponin derived from *Panax ginseng*, has not been sufficiently investigated in depression-related experimental models. In this study, we evaluated the antidepressant potential of 20-gluco-Rf in a mouse model established by prefrontal cortex (PFC) infusion of L-alpha-aminoadipic acid (L-AAA), an astrocyte-selective gliotoxin. Oral administration of 20-gluco-Rf, vehicle, or fluoxetine was initiated before L-AAA infusion into the PFC. 20-Gluco-Rf significantly reduced immobility time in the forced swim and tail suspension tests compared with vehicle treatment. Furthermore, 20-gluco-Rf improved performance in the novel object recognition test. These behavioral changes were accompanied by reduced mRNA expression of proinflammatory mediators, including IL-1β, IL-6, TNF-α, and lipocalin-2, as well as changes in GFAP and IBA-1 protein expression in the PFC. Collectively, these findings provide preliminary in vivo evidence that 20-gluco-Rf is associated with antidepressant-like and cognition-related behavioral effects in an L-AAA-induced mouse model, accompanied by changes in neuroinflammatory and glial-associated molecular markers.

## 1. Introduction

Major depressive disorder (MDD), a leading cause of disability worldwide, constitutes a critical public health challenge, with depressive disorders affecting more than 330 million individuals globally [1]. Patients commonly experience persistent low mood, anhedonia, cognitive impairment, sleep and appetite disturbances, and suicidal ideation, which collectively have a substantial impact on their overall quality of life and contribute to a significant socioeconomic burden and increased healthcare costs [2,3]. Although depression imposes an annual economic burden of multiple billion dollars, it remains insufficiently treated. Current antidepressants have limitations that, cause adverse effects or low remission rates in approximately 30% of patients [4,5]. Furthermore, prevention is challenging, particularly in vulnerable individuals with multiple chronic conditions [6,7,8]. Previous studies analyzing postmortem brain tissue from patients with MDD and animal models of depression have demonstrated a reduction in astrocyte number in the prefrontal cortex (PFC) [9,10,11,12]. Astrocytes are essential for maintaining the extracellular environment required for neuronal activity, including neuronal differentiation and synaptic efficacy [13,14,15]. Astrocytic abnormalities induce a decrease in the uptake and cycling of glutamate, —the primary excitatory amino acid neurotransmitter that maintains a homeostatic balance with gamma-aminobutyric acid (GABA), leading to its accumulation [7]. This may ultimately contribute to the development of depression and can be identified by changes in astrocytic markers, such as glial fibrillary acidic protein (GFAP).

Recent investigations have demonstrated that pharmacological interventions capable of restoring astrocytic function, including the attenuation of neuroinflammatory and glutamatergic disturbances, are associated with antidepressant-like effects in animal models. Since astrocytic dysfunction is closely linked to the amplification of neuroinflammatory cascades, restoration of astrocytic integrity has been proposed as a potential therapeutic strategy for depression. In this context, administration of L-alpha-aminoadipic acid (L-AAA), an astrocyte-selective gliotoxin, induces targeted astrocytic dysfunction and consequent behavioral alterations, thereby providing an experimental paradigm for evaluating astrocyte-targeted antidepressant interventions [7,16].

Previous studies have reported that neuroinflammation contributes to depression pathophysiology. Interleukin(IL)-1β, IL-6, and tumor necrosis factor (TNF)-α are considered key inflammatory mediators that exacerbate depressive pathology [17], and elevated levels of these cytokines have been observed in the hippocampus and prefrontal cortex in animal models of depression [18]. In addition, astrocytes influence the expression of a wide range of inflammatory cytokines. Lipocalin-2 (LCN2), a pro-inflammatory cytokine mediator, has been associated with depression and is often accompanied by increased levels of IL-1β, IL-6, and TNF-α [19,20] and astrocyte-derived LCN2 is thought to be involved in the development of neuroinflammation [21]. Increased expression of LCN2 has been observed in stress-induced animal models of depression as well as in human patients [20,22,23,24,25]. Therefore, drugs that regulate neuroinflammatory markers, including LCN2, IL-1β, IL-6, and TNF-α, as well as astrocytic pathology, may hold promise for alleviating depressive symptoms.

*Panax ginseng* Meyer has a long history of use in traditional medicine and has more recently been investigated for its potential effects on mood- and stress-related symptoms. The antidepressant effects of *P. ginseng* extract have been reported in various animal models of depression [26,27,28,29,30]. In addition, several active components and metabolites of *P. ginseng*, including Rb3 [31], compound K [28], Rb1 [28], 20(S)-protopanaxadiol [32], and Rg1 [33,34], have been shown to ameliorate depression-like behaviors in rodents, suggesting that the bioactive constituents of *P. ginseng* may exert meaningful therapeutic effects in MDD.

Among more than 50 ginsenosides identified in *P. ginseng*, 20-glucoginsenoside Rf (20-gluco-Rf) is a steroidal glycoside classified as a protopanaxatriol (PPT)-type ginsenoside [35]. Although 20-gluco-Rf is present only in trace amounts among ginsenosides, it is a bioactive saponin constituent of *P. ginseng* [36]. Its saponin structure has been characterized, and it belongs to the triol-type ginsenosides, in which sugar moieties are attached to hydroxyl groups at C-6 and C-20 [37]. Importantly, several structurally related PPT-type triol saponins—such as ginsenosides Re [38], Rg2 [39], and Rf [40]—have been shown to exert antidepressant-like effects in rodent models. Because 20-gluco-Rf belongs to the same PPT-type triol saponin family as these bioactive ginsenosides, it is reasonable to propose that it may also possess biological relevance in depression, particularly in pathways involving glial or inflammatory regulation. Previous studies on other ginsenosides suggest that ginsenosides may have therapeutic relevance to MDD, a representative neuropsychiatric disorder. Although several structurally related ginsenosides have demonstrated antidepressant and anti-inflammatory properties, the pharmacological actions of 20-gluco-Rf remain largely unknown. In particular, it is not yet known whether 20-gluco-Rf influences astrocytic integrity and neuroinflammatory signaling, which are key biological processes implicated in the pathophysiology of MDD. This limited understanding underscores the need for a systematic investigation into the neurobiological effects of 20-gluco-Rf.

Accordingly, we hypothesized that 20-gluco-Rf may be associated with antidepressant-like behavioral effects and changes in neuroinflammatory and glial-associated molecular markers in the PFC. To test this hypothesis, we evaluated behavioral outcomes, inflammatory gene expression, and glial-associated protein expression in an L-AAA-induced mouse model.

## 2. Materials and Methods

### 2.1. Animals

Eight-week-old male C57BL/6 mice (Young Bio, Inc., Seongnam, Gyeonggi-do, Republic of Korea) weighing 20–22 g were used. The mice were housed in acrylic cages (26 × 42 × 18 cm) under automatically monitored and controlled temperature (22 ± 2 °C) and relative humidity (60 ± 10%), with free access to food and water, and maintained on a 12-h light/dark cycle. All behavioral tests were conducted between 9:00 and 17:00. All the procedures were approved by the Kyung Hee University Medical Center Institutional Animal Care and Use Committee (approval number: KHMC-IACUC 20-021-01).

### 2.2. Drug Administration

**Figure 1:**
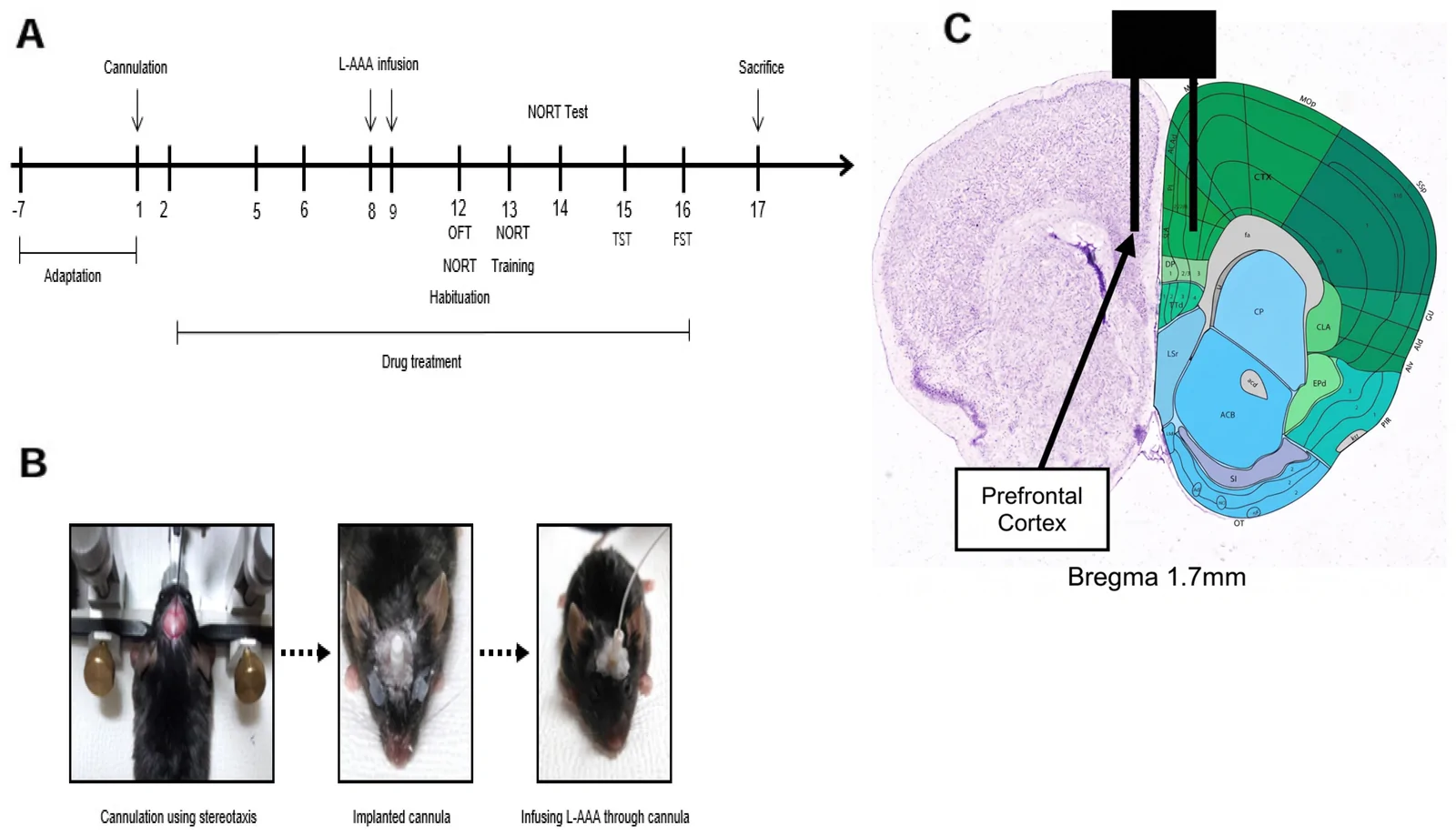
(**A**) Experimental timeline. (**B**) Schematic representation of cannula implantation. After the implantation of guide cannulas, L-AAA was infused bilaterally at 0.1 μL/min for 6 min per hemisphere (0.6 μL per hemisphere; 1.2 μL per mouse per administration). (**C**) Location of L-AAA infusion. The guide cannula was aimed at the prefrontal cortex using the following coordinates: AP 1.7 mm, ML ±0.3 mm, and DV −2.5 mm from bregma. Allen Mouse Brain Atlas, atlas.brain-map.org [46] (accessed on 1 July 2026) and mouse.brain-map.org [47] (accessed on 1 July 2026). L-AAA, L-alpha-aminoadipic acid; TST, tail suspension test; FST, forced swim test; OFT, open-field test; NORT, novel object recognition test; AP, anterior–posterior; ML, medial–lateral; DV, dorsal–ventral.

Mice were randomly allocated to one of four experimental groups: (i) Sham control (Sham), distilled water per os (po) (n = 10); (ii) L-AAA (100 mg/mL) + vehicle (Veh), distilled water po (n = 10); (iii) L-AAA + fluoxetine (10 mg/kg) po (n = 10); and (iv) L-AAA + 20-gluco-Rf (20 mg/kg) po (n = 10). The Sham group did not undergo anesthesia or any surgery-related procedure, including scalp incision, skull drilling, stereotaxic manipulation, cannula implantation, or intracerebral infusion. Animals were initially assigned at n = 10 per group. However, the final sample size differed among behavioral and molecular analyses. This difference was due to postoperative mortality, incomplete behavioral recording, and insufficient tissue, RNA, or protein quality. In addition, tissue samples were allocated to different molecular assays. The final n values for each experiment are provided in the corresponding figure legends. 20-Glucoginsenoside Rf (20-gluco-Rf; CAS No. 68406-27-9; catalog No. CFN95036; purity ≥ 98%) was purchased from ChemFaces (Wuhan, Hubei, China) and is reported by the supplier to be a natural product derived from the roots of *Panax ginseng* C. A. Mey. The dose of 20-gluco-Rf (20 mg/kg) was selected as an exploratory dose based on previous in vivo studies using structurally related ginsenosides [41,42,43]. However, because the dose–response and pharmacokinetic profiles of 20-gluco-Rf have not yet been established, this dose was considered exploratory rather than optimized. The overall experimental schedule is shown in Figure 1A. 20-gluco-Rf and fluoxetine were prepared on a clean bench using sterilized distilled water and orally administered to the mice at a volume of 200 μL per mouse via oral gavage using gavage needles. Vehicle-treated mice received the same volume of sterilized distilled water. All administration equipment, including oral gavage needles and syringes, was sterilized with ultraviolet light prior to use. The fluoxetine dose of 10 mg/kg was selected as a reference antidepressant comparator dose with reference to previous mouse studies reporting behavioral efficacy at this dose in rodent models of depression [44,45].

### 2.3. Cannula Implantation and Injection of L-AAA

Mice in the three L-AAA-treated groups were anesthetized by intraperitoneal injection of a mixture of Zoletil 50 (Virbac Korea Co., Ltd., Seoul, Republic of Korea; 50 mg/mL), Rompun (xylazine 20 mg/mL), and sterile saline at a ratio of 5:2:3 (*v*/*v*/*v*). Each mouse received 25 μL of the anesthetic mixture. For a mouse weighing approximately 25 g, this corresponded to approximately 25 mg/kg Zoletil and 4 mg/kg xylazine. The mice were then placed in a stereotaxic apparatus (Vernier Stereotaxic Instrument; Leica Biosystems, Nussloch, Germany). A guide cannula (RWD Life Science Co., Ltd., Shenzhen, Guangdong, China) was implanted into the PFC using the following coordinates: 1.7 mm anterior–posterior (AP), ±0.3 mm medial–lateral (ML), and −2.5 mm dorsal–ventral (DV) from bregma. Following cannula implantation, no postoperative analgesic was administered, in accordance with the approved IACUC protocol. After surgery, the mice were placed on a heating pad maintained at approximately 35 °C and monitored for 30–40 min until recovery from anesthesia. Thereafter, the animals were monitored daily for signs of infection, distress, abnormal behavior, and changes in body weight. All animals were allowed to recover for seven days before L-AAA infusion.

After one week of recovery, L-AAA (100 mg/mL, equivalent to 100 μg/μL; Sigma-Aldrich, St. Louis, MO, USA) was infused bilaterally into the PFC at a rate of 0.1 μL/min for 6 min per hemisphere (0.6 μL per hemisphere; 60 μg per hemisphere), resulting in a total dose of 120 μg per mouse per administration. The infusion was administered once daily for two consecutive days.

After cannula implantation, mice were maintained on a warming pad until recovery from anesthesia and monitored daily for signs of infection, distress, abnormal behavior, or changes in body weight. Routine prophylactic antibiotics were not administered unless signs of infection were observed, in accordance with the approved animal experimental protocol.

Cannula were stereotaxically targeted to the PFC using the coordinates AP +1.7 mm, ML ±0.3 mm, and DV −2.5 mm from bregma. Cannula placement was not independently verified by postmortem histological examination.

### 2.4. Behavioral Tests

All behavioral tests were conducted under controlled environmental conditions. The open-field test (OFT) and novel object recognition test (NORT) were performed in a dark, quiet room. The tail suspension test (TST) and forced swim test (FST) were performed in a bright room, with mice visually separated from each other during testing to prevent behavioral influence between animals.

#### 2.4.1. OFT

The OFT was performed in a dark, quiet room. The apparatus consisted of four acrylic boxes (50 × 50 × 50 cm), and a video camera was installed above the apparatus to record mouse activity. Each mouse (n = 9–10 per group) was gently placed in the central grid and allowed to explore the apparatus freely. The total distance traveled was analyzed for 10 min using the recorded video files. Other OFT-derived parameters, including activity time, immobility time, time spent in the central or peripheral zones, average travel speed, rearing behavior, and hole-peeping behavior, were not separately analyzed. All data were acquired using video-tracking software (SMART 3.0; Panlab Harvard Apparatus, Barcelona, Spain).

#### 2.4.2. TST

The TST was performed in a bright, quiet room. Mice were attached to adhesive tape approximately one-third of the distance from the tail tip and suspended 50 cm above the floor. Mice were visually separated from each other during testing to prevent behavioral influence between animals. Each mouse (n = 6 per group) was recorded using a camcorder, and immobility time, defined as the duration during which the mouse remained completely motionless while suspended, was measured from the recorded video files during the last 4 min of the 6-min test session. Immobility time was the only TST-derived behavioral parameter analyzed; latency to immobility, escape behavior duration, and the number of immobility episodes were not separately analyzed.

#### 2.4.3. FST

The FST was performed in a bright, quiet room. Mice were individually placed in an open cylindrical container (diameter 20 cm, height 35 cm) filled with water (19 cm depth) maintained at 25 °C and visually separated from each other during testing. Each mouse (n = 6 per group) was recorded using a camcorder, and immobility time, defined as the duration during which the mouse ceased struggling and remained floating motionless, was measured from the recorded video files during the last 4 min of the 6-min test session. Immobility time was the only FST-derived behavioral parameter analyzed; struggling time and swimming time were not separately quantified or analyzed.

#### 2.4.4. NORT

The NORT was conducted in an open-field arena containing two different types of objects. Both objects were comparable in height and volume but differed in shape and appearance. During the 5-min habituation period, conducted 2 days before testing, the animals were allowed to explore the empty arena. After 24 h, the animals were exposed for 10 min to a familiar arena containing two identical objects placed at equal distances. On the following day (test day), the mice were allowed to explore the arena for 10 min in the presence of one familiar object and one novel object to assess long-term recognition memory. The NORT was performed under the same dark and quiet conditions as the OFT. Mouse behavior was recorded using a video camera installed above the apparatus. Each mouse (n = 5 per group) was allowed to explore the objects freely, and total exploration time and the discrimination index were analyzed using the recorded video files. All data were acquired using video-tracking software (SMART 3.0; Panlab Harvard Apparatus, Barcelona, Spain).

For the present analysis, total exploration time was defined as the sum of the time spent in the quadrants containing the familiar and novel objects. The discrimination index was calculated as [Tnovel/(Tnovel + Tfamiliar)] × 100, where Tnovel and Tfamiliar represent the time spent in the quadrants containing the novel and familiar objects, respectively. No minimum exploration-time criterion was prespecified, and no animals were excluded because of insufficient exploration. The identities of the novel and familiar objects were not counterbalanced, and the left/right position of the novel object was not counterbalanced. After each trial, the arena and objects were cleaned with 70% ethanol and allowed to air for approximately 2 min before the next animal was tested. During behavioral testing, the experimenter remained outside the animal’s visual field. Formal blinding to the experimental groups was not performed during video analysis.

### 2.5. Tissue Collection

After completion of the behavioral tests, all mice were anesthetized and euthanized. The PFC was rapidly dissected from each mouse. Bilateral PFC tissues from each mouse were pooled and used as a single sample for subsequent real-time PCR and Western blot analyses. The collected PFC tissues were immediately frozen in liquid nitrogen and subsequently stored at −80 °C until further analysis. Tissue samples designated for Western blot analysis were used for protein extraction, whereas samples designated for real-time PCR analysis were processed for total RNA extraction using TRIzol reagent according to the manufacturer’s instructions. For tissue collection, mice were anesthetized by intraperitoneal injection of 300 μL of a mixed anesthetic solution consisting of Zoletil, xylazine, and sterile saline at a ratio of 5:2:3. After deep anesthesia was confirmed, mice were euthanized by cervical dislocation, and the prefrontal cortex tissues were rapidly collected for subsequent molecular analyses.

### 2.6. Western Blot Analysis

The PFC tissue was homogenized in 1× RIPA buffer (Pierce Biotechnology, Rockford, IL, USA). The homogenates were centrifuged at 12,000× *g* for 15 min at 4 °C, after which the supernatants were obtained. Protein concentrations were determined using the BCA protein assay kit (Pierce Biotechnology, Inc., Rockford, IL, USA). Equal amounts of protein (30 μg per lane) were separated on 12% sodium dodecyl sulfate–polyacrylamide gel electrophoresis gels and transferred onto polyvinylidene fluoride membranes (Millipore, Burlington, MA, USA). The membranes were incubated overnight at 4 °C with primary antibodies diluted 1:1000 in 1% BSA solution: mouse monoclonal anti-GFAP (3670S, Cell Signaling Technology, Danvers, MA, USA) and rabbit polyclonal anti-IBA-1 (PA5-27436, Invitrogen, Carlsbad, CA, USA). After washing, the membranes were incubated with the appropriate HRP-conjugated secondary antibodies (1:5000) for 1 h at room temperature. The immunoreactive bands were visualized using an enhanced chemiluminescence kit (34577, Thermo Fisher Scientific, Waltham, MA, USA) and imaged using the Amersham Imager 600 (GE Healthcare Bio-Sciences AB, Uppsala, Sweden). Band intensities were quantified using ImageJ software (version 1.44; National Institutes of Health, Bethesda, MD, USA).

### 2.7. Quantitative Real-Time PCR

Total RNA was extracted from mouse PFC tissue using TRIzol reagent (Cat. No. 15596026; Thermo Fisher Scientific, Waltham, MA, USA) according to the manufacturer’s instructions. For each sample, 1 μg of total RNA was treated with 1 U of DNase I prior to reverse transcription. First-strand cDNA was synthesized using the RevertAid First Strand cDNA Synthesis Kit (Cat. No. K1622; Thermo Scientific, Waltham, MA, USA) with an oligo(dT)18 primer according to the manufacturer’s protocol. The reverse-transcription reaction was performed in a total volume of 20 μL at 42 °C for 60 min, followed by enzyme inactivation at 70 °C for 5 min. The nucleotide sequences of primers were based on previously published cDNA se-quences (Table 1).

Quantitative real-time PCR was performed using an Applied Biosystems StepOnePlus™ Real-Time PCR System with Power SYBR™ Green PCR Master Mix (Cat. No. 4367659; Applied Biosystems/Thermo Fisher Scientific, Waltham, MA, USA). Each reaction was performed in a total volume of 20 μL containing 1 μL of cDNA template, 1 μL of forward primer, and 1 μL of reverse primer. Each sample was analyzed in technical triplicate. PCR amplification was performed for 40 cycles using the gene-specific primers listed in Table 1, with an annealing/extension temperature of 60 °C. Melting-curve analysis was automatically performed by the StepOnePlus system following amplification.

Relative gene expression was calculated using the ΔΔCt method, with GAPDH as the endogenous reference gene and the Sham group as the calibrator.

### 2.8. Statistical Analysis

All data are presented as the mean ± SEM. Statistical analyses were performed using GraphPad Prism software (version 8.0.1; GraphPad Software, San Diego, CA, USA). Data distribution was assessed before group comparisons. Differences among groups were analyzed using one-way analysis of variance (ANOVA), followed by Tukey’s multiple-comparison test. A value of *p* < 0.05 was considered statistically significant.

## 3. Results

### 3.1. Effects of 20-Gluco-Rf on Depression-like Behavior in Mice Injected with L-AAA

#### 3.1.1. OFT

**Figure 2:**
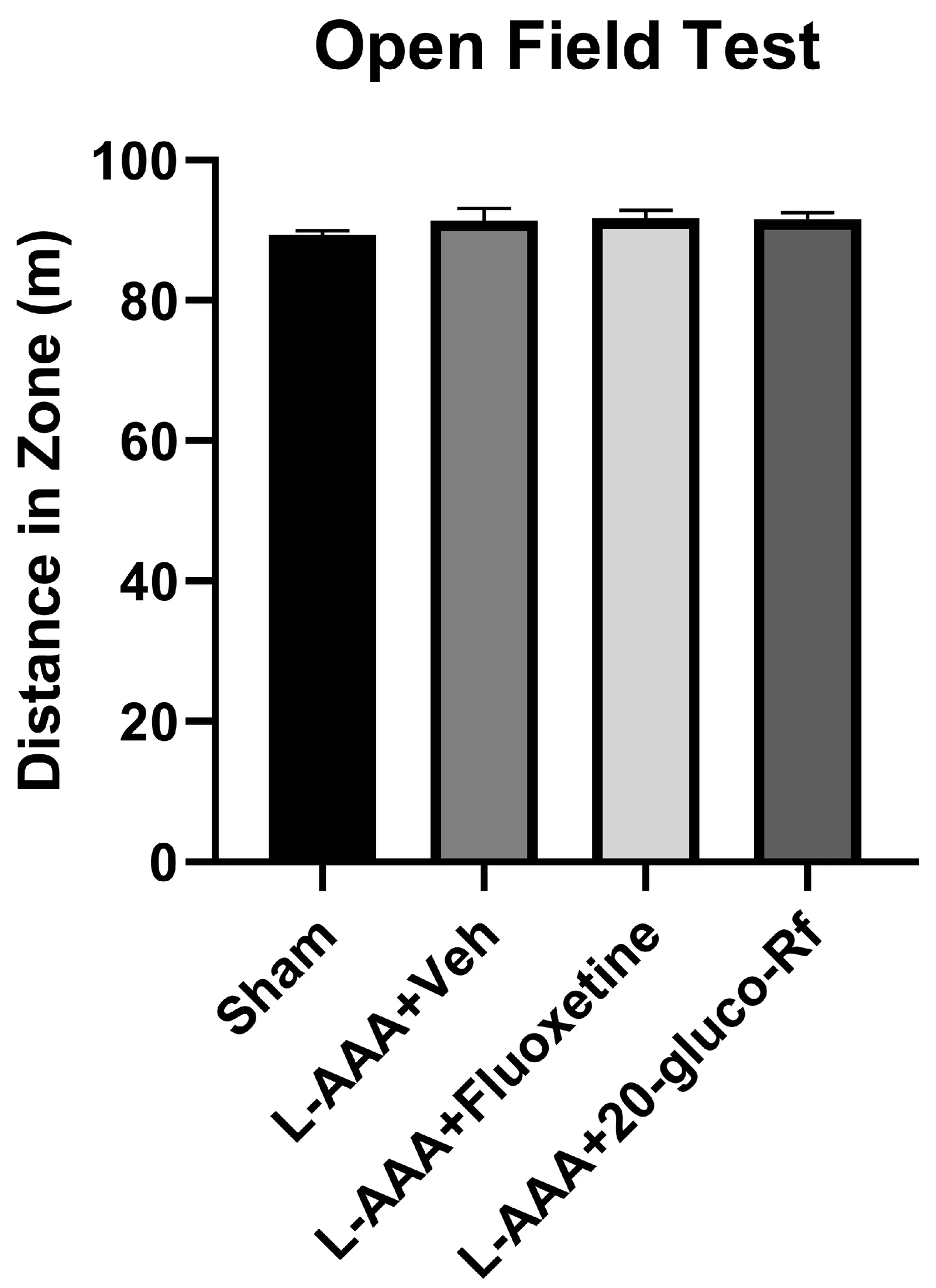
Effect of 20-gluco-Rf on the ost in mice injected with L-alpha-aminoadipic acid into the prefrontal cortex. Locomotor activity was assessed by the total distance traveled across the entire arena, and no significant difference was observed among groups. L-AAA, L-alpha-aminoadipic acid; OFT, open-field test.

First, we evaluated whether L-AAA affected locomotor activity in each group. There was no statistically significant difference in total ambulatory distance between the groups in the open-field test (OFT) (*p* > 0.05) (Figure 2).

#### 3.1.2. TST

**Figure 3:**
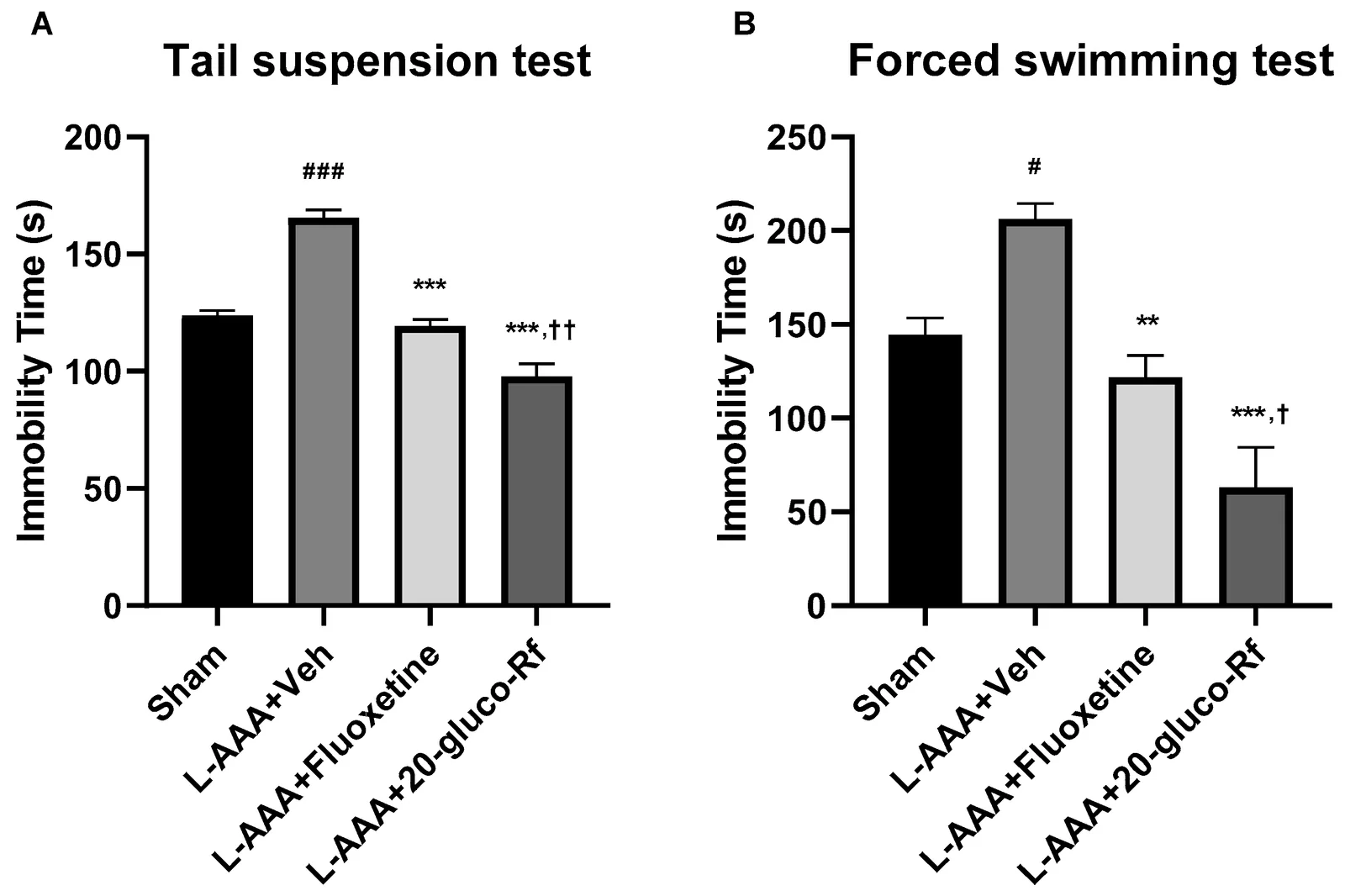
Effects of 20-gluco-Rf on immobility time in the TST and FST. The immobility time in (**A**) TST and (**B**) FST was measured to evaluate depression-like behavior in mice infused with L-AAA into the prefrontal cortex. L-AAA + Veh mice exhibited significantly increased immobility compared with the Sham group (# *p* < 0.05 for FST; ### *p* < 0.001 for TST). Oral administration of fluoxetine (10 mg/kg) or 20-glucoginsenoside Rf (20-gluco-Rf; 20 mg/kg) significantly reduced immobility relative to the L-AAA + Veh group. Results are presented as mean ± SEM (n = 6 per group). # *p* < 0.05, and ### *p* < 0.001 vs. Sham group; ** *p* < 0.01, and *** *p* < 0.001 vs. L-AAA + Veh group; † *p* < 0.05, and †† *p* < 0.01 between the fluoxetine and 20-gluco-Rf groups. L-AAA, L-alpha-aminoadipic acid; Veh, vehicle; TST, tail suspension test; FST, forced swim test; SEM, standard error of the mean.

The tail suspension test (TST) was performed to evaluate the antidepressant-like effects of 20-gluco-Rf in mice injected with L-AAA. In the TST, 20-gluco-Rf-treated mice spent significantly less time immobile than did the vehicle-treated group (*p* < 0.01), and fluoxetine-treated mice showed a significant reduction in immobility time (*p* < 0.01). In contrast, immobility time was significantly increased in the vehicle-treated mice compared to that in the sham group (*p* < 0.01, Figure 3A). This result shows that 20-gluco-Rf treatment reduced immobility time in the TST in L-AAA-infused mice.

#### 3.1.3. FST

The forced swim test (FST) was performed to evaluate the antidepressant-like effects of 20-gluco-Rf in mice injected with L-AAA. In the FST, mice treated with 20-gluco-Rf spent significantly less time immobile than did the vehicle-treated group (*p* < 0.05), and fluoxetine-treated mice demonstrated reduced immobility time (*p* < 0.05). In contrast, vehicle-treated mice showed a significantly increased immobility time than did the sham group (*p* < 0.05, Figure 3B). This result shows that 20-gluco-Rf treatment reduced immobility time in the FST in L-AAA-infused mice.

#### 3.1.4. NORT

**Figure 4:**
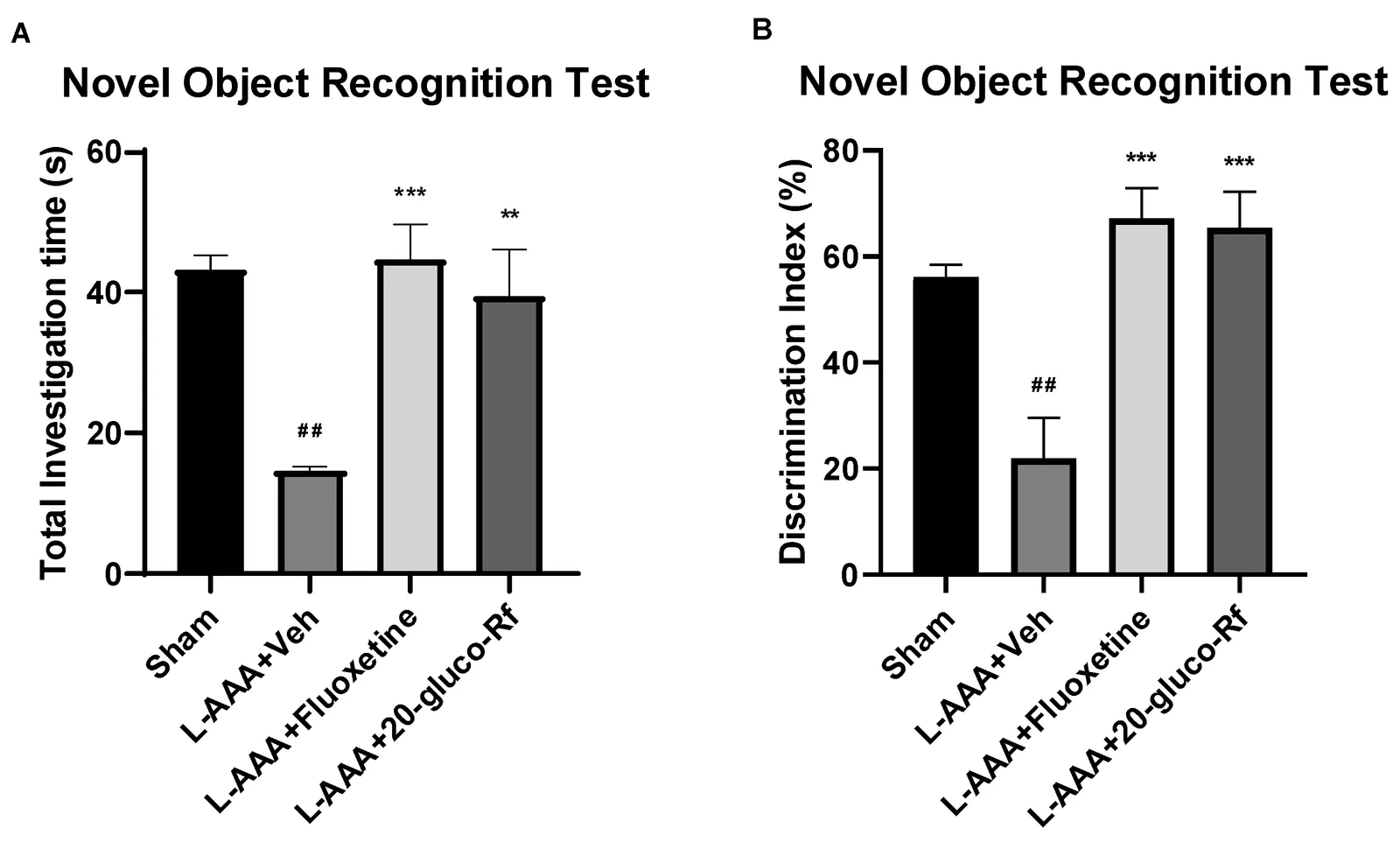
Effects of 20-gluco-Rf on recognition memory in the NORT. (**A**) Total exploration time, defined as the sum of the time spent in the quadrants containing the familiar and novel objects. L-AAA + Veh mice showed reduced exploration compared with the Sham group (## *p* < 0.01), whereas fluoxetine and 20-gluco-Rf treatment significantly increased exploration time compared with the L-AAA + Veh group (** *p* < 0.01; *** *p* < 0.001). (**B**) Discrimination index, calculated as [Tnovel/(Tnovel + Tfamiliar)] × 100, where Tnovel and Tfamiliar represent the time spent in the quadrants containing the novel and familiar objects, respectively. L-AAA + Veh mice displayed a reduced discrimination index compared with the Sham group (## *p* < 0.01), whereas fluoxetine- and 20-gluco-Rf–treated mice exhibited significantly higher discrimination indices than the L-AAA + Veh group (*** *p* < 0.001). Results are presented as mean ± SEM (n = 5 per group). L-AAA, L-alpha-aminoadipic acid; Veh, vehicle; NORT, novel object recognition test; SEM, standard error of the mean.

Novel object recognition test (NORT) was performed to evaluate the effects of 20-gluco-Rf on the cognitive performance of mice injected with L-AAA. The total exploration time was significantly decreased in the vehicle-treated mice compared to that in the sham group (*p* < 0.01). In contrast, 20-gluco-Rf–treated and fluoxetine-treated mice displayed an increased total exploration time comparable to that of the sham group (*p* < 0.01, Figure 4A). Similarly, the discrimination index was significantly lower in vehicle-treated mice than in the sham group (*p* < 0.01). In contrast, 20-gluco-Rf–treated and fluoxetine-treated mice exhibited a significantly increased discrimination index compared to the vehicle-treated group (*p* < 0.01, Figure 4B). These results show that 20-gluco-Rf treatment was associated with an increased discrimination index in L-AAA-infused mice.

### 3.2. Effects of 20-Gluco-Rf on Proinflammatory Cytokine and LCN2 mRNA Expression in the PFC

**Figure 5:**
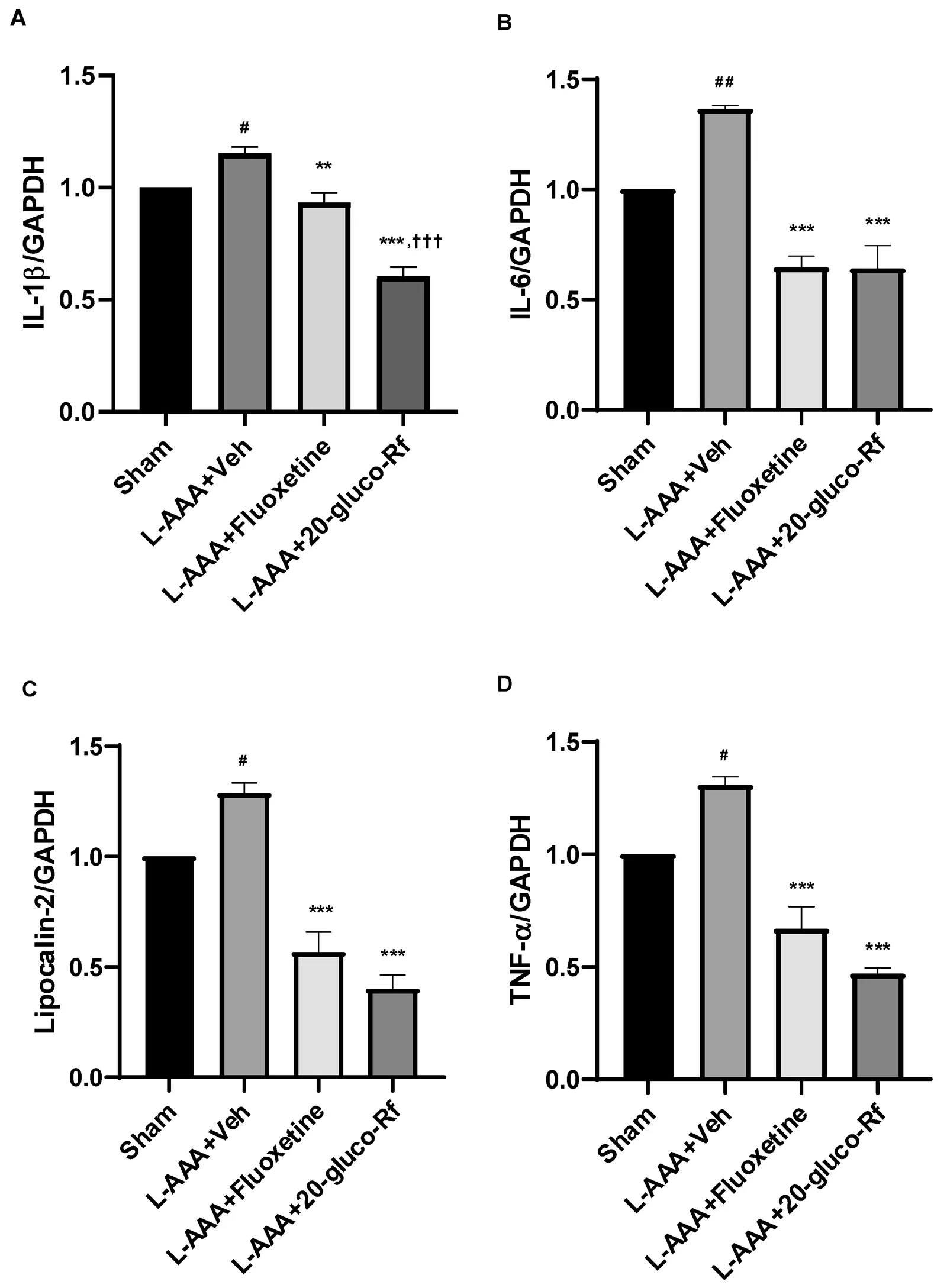
Quantitative real-time PCR analysis of (**A**) IL-1β, (**B**) IL-6, (**C**) LCN2 and (**D**) TNF-α mRNA expression in the PFC of mice infused with L-AAA. L-AAA + Veh mice exhibited significantly increased expression of all inflammatory markers and LCN2 compared with that in the Sham group (# *p* < 0.05; ## *p* < 0.01). Oral administration of fluoxetine (10 mg/kg) or 20-gluco-Rf (20 mg/kg) reduced cytokine and LCN2 mRNA levels relative to the L-AAA + Veh group. Expression levels were normalized to GAPDH. Results are presented as the mean ± SEM (n = 5 per group). # *p* < 0.05, and ## *p* < 0.01 vs. Sham group; ** *p* < 0.01, and *** *p* < 0.001 vs. L-AAA + Veh group; ††† *p* < 0.001 between the fluoxetine and 20-gluco-Rf groups. L-AAA, L-alpha-aminoadipic acid; Veh, vehicle; PFC, prefrontal cortex; LCN2, lipocalin-2; SEM, standard error of the mean.

Quantitative real-time PCR was performed to assess proinflammatory cytokine and LCN2 mRNA expression in the PFC. The mRNA expression levels of IL-1β and TNF-α were significantly increased in the L-AAA + Veh group compared with the Sham group. In contrast, 20-gluco-Rf-treated mice showed significantly decreased IL-1β and TNF-α mRNA expression relative to the L-AAA + Veh group (*p* < 0.05), and fluoxetine-treated mice also exhibited reduced expression of these markers (*p* < 0.05; Figure 5A,D).

Similarly, IL-6 mRNA expression was significantly higher in the L-AAA + Veh group than in the Sham group. In contrast, 20-gluco-Rf- and fluoxetine-treated mice showed significantly decreased IL-6 mRNA expression relative to the L-AAA + Veh group (*p* < 0.05; Figure 5B).

LCN2 mRNA expression was significantly higher in the L-AAA + Veh group than in the Sham group. In contrast, 20-gluco-Rf-treated mice showed significantly decreased LCN2 mRNA expression compared with the L-AAA + Veh group (*p* < 0.05), and fluoxetine-treated mice also exhibited reduced LCN2 mRNA expression (*p* < 0.05; Figure 5C). Collectively, these results indicate that 20-gluco-Rf treatment was associated with reduced inflammatory-marker mRNA expression in the PFC.

### 3.3. Effects of 20-Gluco-Rf on GFAP and IBA-1 Protein Expression in the PFC

Evidence suggests that molecular alterations localized to a specific brain area can be associated with functional changes in interconnected neural pathways [48,49]. In the pathology of depression, the PFC, hippocampus, and amygdala are critical substrates [50]. Notably, the behavioral phenotypes induced by L-AAA-induced glial-associated alterations are highly region-specific: whereas prelimbic cortex infusion is primarily linked to affective deficits such as apathy and anhedonia, and targeting the hippocampal region predominantly impairs learning functions [51].

**Figure 6:**
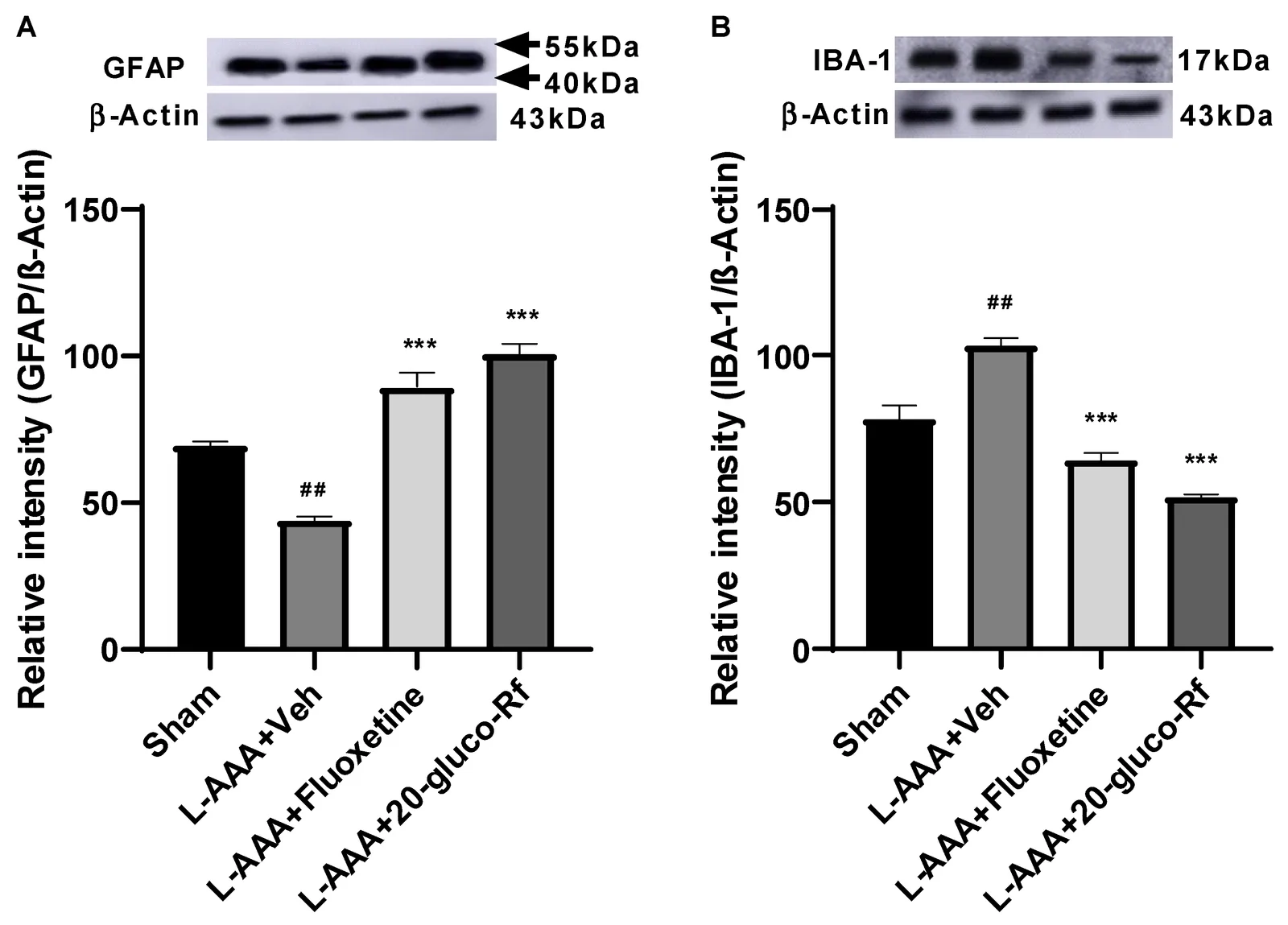
(**A**) GFAP/β-actin relative expression: Western blot analysis of GFAP/β-actin in mouse PFC tissues of Sham, L-AAA + Veh, L-AAA + Fluoxetine, and L-AAA + 20-gluco-Rf. (**B**) IBA-1/β-actin relative expression: Western blot analysis of IBA-1/β-actin in mouse PFC tissues of Sham, L-AAA + Veh, L-AAA + Fluoxetine, and L-AAA + 20-gluco-Rf. Data represent means ± SEM. ## *p* < 0.01 compared with Sham; *** *p* < 0.0001 compared with the L-AAA + Veh group. L-AAA, L-alpha-aminoadipic acid; Veh, vehicle; PFC, prefrontal cortex; GFAP, glial fibrillary acidic protein; IBA-1, ionized calcium-binding adaptor molecule 1; SEM, standard error of the mean.

GFAP protein expression was significantly reduced in the L-AAA + Veh group, suggesting L-AAA-induced disruption of astrocyte-associated marker expression in the PFC (Figure 6A). Conversely, the expression of IBA-1 expression was increased in the L-AAA-infused mice (## *p* < 0.01), suggesting IBA-1-associated microglial marker changes in the PFC (Figure 6B).

Oral administration of 20-gluco-Rf significantly increased GFAP protein levels compared to those in the L-AAA + Veh group (*** *p* < 0.0001) (Figure 6A). Furthermore, 20-gluco-Rf treatment reduced the L-AAA-induced increase in IBA-1 expression (*** *p* < 0.0001) (Figure 6B). These effects were comparable to those observed in the fluoxetine-treated group. These findings indicate that 20-gluco-Rf was associated with GFAP- and IBA-1-associated protein-expression changes in the PFC.

## 4. Discussion

The present study examined the effects of 20-gluco-Rf on behavioral alterations and inflammatory and glial-associated molecular markers in an L-AAA-induced mouse model. Previous postmortem human studies have consistently reported abnormalities in astrocyte function, density, and morphology in patients with mood disorders [9,10,11,12,52,53]. Based on this evidence, we focused on astrocyte-associated dysfunction as a pathological feature relevant to depression and employed an L-AAA-induced model, in which an astrocyte-selective gliotoxin was infused into the PFC. In this model, 20-gluco-Rf treatment was associated with reduced depression-like behavioral responses and improved NORT performance. These behavioral changes were accompanied by reduced inflammatory marker expression and changes in GFAP and IBA-1 protein expression. Overall, these findings suggest that 20-gluco-Rf may have pharmacological relevance in L-AAA-induced behavioral alterations accompanied by neuroinflammatory and glial-associated molecular changes.

Despite the growing interest in the antidepressant potential of ginsenosides [30,54], evidence regarding 20-gluco-Rf in animal models of depression remains largely unexplored. Notably, earlier studies on other ginsenosides predominantly focused on chronic stress paradigms rather than astrocyte-targeted models. Previous postmortem studies and recent reviews have reported reduced astrocyte density or altered astrocyte-related markers in mood-related brain regions, including the PFC and hippocampus [9,10,11,12,52]. Accordingly, we utilized an astrocyte-ablation model based on L-AAA, a selective gliotoxin. The infusion of L-AAA into the PFC induces transient astrocytic ablation and glial degeneration, thereby providing an experimental model for examining astrocyte-related mechanisms relevant to depression [7,55,56]. Thus, L-AAA infusion into the PFC is regarded as an astrocytic dysfunction-based model of depression [57,58,59]. As a glutamate analog, L-AAA preferentially affects astrocytes and disrupts astrocyte-related functions, providing a useful experimental approach for examining glial-associated changes [55,56]. Several studies have used this model to evaluate the antidepressant efficacy of various compounds [7,60]. GFAP was selected as the primary astrocyte-associated marker because it remains widely used for characterizing astrocytic alterations in post-mortem brains of patients with MDD [52,61,62]. Consequently, we employed GFAP to quantify the protein-level changes within the PFC following the administration of 20-gluco-Rf in our L-AAA-induced ablation model.

The TST and FST, which are widely used to assess depression-like behaviors in rodents, are well established as sensitive to antidepressant treatment [63,64,65]. Increased immobility in these paradigms represents an antidepressant-sensitive behavioral response. In the present study, L-AAA–injected mice exhibited prolonged immobility. Notably, both fluoxetine and 20-gluco-Rf reduced immobility in both assays relative to the L-AAA + Veh group, suggesting antidepressant-like behavioral effects under the present experimental conditions; however, the present study was not designed or powered to determine the relative efficacy of 20-gluco-Rf versus fluoxetine. Therefore, these findings should not be interpreted as evidence of superiority over fluoxetine. In addition to mood-related symptoms, cognitive impairment is recognized as an important pathological feature of MDD, although most currently available treatments primarily target affective dysregulation [66,67,68,69]. Patients with depression frequently exhibit memory, attention, and cognitive flexibility deficits, which can be evaluated in animal models using a range of behavioral tests [70]. In this context, 20-gluco-Rf also improved NORT performance, as evidenced by increased exploration and a higher discrimination index for the novel object. These findings suggest that 20-gluco-Rf may influence both the affective-like and cognitive behavioral domains relevant to MDD.

The mRNA expression levels of IL-1β, IL-6, and TNF-α were increased following L-AAA exposure, whereas 20-gluco-Rf administration reduced their mRNA expression in the PFC. Neuroinflammatory processes have also been linked to alterations in serotonin and glutamate signaling relevant to depression [71]. Thus, attenuation of neuroinflammatory marker expression may be one biological process associated with antidepressant-like behavioral effects in this model. IL-1β, IL-6, and TNF-α are regarded as key inflammatory mediators that exacerbate depressive pathology [17]. Meta-analytic data indicate that fluoxetine reduces these cytokines [72], and tricyclic antidepressants suppress their expression [73]. Upregulation of inflammatory markers has been consistently observed in several animal models of MDD [18]. Moreover, IL-1β knockout, IL-6 knockout, and TNF-α receptor knockout mice exhibit resistance to depression induction [74,75,76,77,78]. In line with these findings, we measured the mRNA expression of proinflammatory cytokines as inflammatory markers. L-AAA increased IL-1β, IL-6, and TNF-α mRNA expression, whereas 20-gluco-Rf reduced their expression.

Additionally, expression of LCN2, a major astrocyte-regulated inflammatory mediator, was reduced in the 20-gluco-Rf–treated group. Previous studies have shown that LCN2 functions as an inflammatory modulator of cytokine induction and inflammatory cell recruitment [79]. In the present study, LCN2 expression was measured as an additional index to evaluate the antidepressant potential of 20-gluco-Rf. LCN2 is known to induce inflammatory gene expression, including IL-1β, IL-6, and TNF-α [19,21]. Moreover, because LCN2 remains stably elevated regardless of antidepressant use or episode timing, it has been proposed as a putative biomarker of depression [25]. Elevated LCN2 levels are also associated with depression-like behavioral changes in ovariectomized rodents [23,80]. Considering that astrocyte-related alterations are linked to depression pathophysiology, LCN2, which is regulated in part by astrocytes, may contribute to inflammatory processes associated with depressive states [81,82,83]. Collectively, these behavioral and molecular findings suggest that 20-gluco-Rf may be associated with antidepressant-like behavioral effects accompanied by changes in astrocyte-associated markers and LCN2-related inflammatory responses.

Western blot analysis demonstrated that the L-AAA-induced reduction in GFAP and the concomitant increase in IBA-1 expression in the PFC were significantly attenuated by 20-gluco-Rf treatment. These molecular changes aligned with the observed improvements in FST, TST, and NORT. Considering that astrocytic dysfunction and the subsequent neuroinflammatory responses are central to the pathophysiology of depression [9,10,52], our findings suggest that 20-gluco-Rf may be associated with changes in the neuroglial marker profile in the PFC under gliotoxic conditions. Because GFAP and IBA-1 were assessed by Western blotting, these findings should be interpreted as protein-level marker changes rather than as direct morphological evidence of glial structural alterations.

First, the present study was designed as an initial in vivo pharmacological evaluation of 20-gluco-Rf, and pathway-specific assays were not performed. Therefore, the data do not identify the molecular signaling events underlying the observed behavioral and molecular changes. Further mechanistic studies will be required before a causal pathway can be established.

Second, several features of the experimental design constrain the generalizability of the findings. Only a single exploratory dose of 20-gluco-Rf was evaluated; consequently, the dose–response relationship, optimal therapeutic dose, and dose-dependent pharmacological profile of the compound remain undefined, and the selected dose, derived from studies of structurally related ginsenosides, cannot be regarded as optimized. In addition, because L-AAA-induced astrocytic ablation persists for approximately three days [55], a compressed paradigm was used to capture short-term effects, so the sustained efficacy of 20-gluco-Rf under chronic conditions remains untested. The design also lacked a cannula-implanted, saline-infused surgical control. Because the Sham group underwent neither stereotaxic surgery nor intracerebral infusion, differences between the Sham and L-AAA-treated groups cannot be attributed exclusively to L-AAA, as possible effects of the surgical and infusion procedures cannot be excluded. In addition, because cannula placement was not histologically verified in individual animals, variability in the precise location of L-AAA delivery cannot be excluded. A dedicated toxicity assessment of 20-gluco-Rf was not performed in the present study; therefore, the current findings do not establish the safety profile of 20-gluco-Rf at the tested dose. Finally, although statistically significant differences were detected across several outcomes, the modest final sample sizes limit statistical power. Future work should incorporate multiple dose levels, a saline-infused surgical control group, chronic and ethologically relevant stress paradigms such as chronic unpredictable stress [84], and larger cohorts informed by an a priori power calculation.

Third, the level at which the outcomes were measured limits the inferences that can be drawn. GFAP and IBA-1 were quantified by Western blotting, and the results should therefore be interpreted as changes in glial marker expression rather than as direct morphological evidence of astrocyte protection, microglial activation, or structural restoration; immunohistochemical or immunofluorescence analysis with morphometric quantification would be required to determine whether these protein-level changes correspond to alterations in glial morphology, cell density, process architecture, or regional distribution within the PFC. Similarly, IL-1β, IL-6, TNF-α, and LCN2 were assessed only at the transcript level, and whether these changes are reflected in protein abundance was not verified; ELISA or western blot analysis is needed for confirmation. Lastly, although identical anesthesia and euthanasia procedures were applied to all groups, anesthetic agents may influence cytokine expression and glial protein levels, and an anesthesia-related contribution to the observed molecular differences cannot be excluded. Future studies should standardize anesthetic exposure and the interval between anesthesia and tissue collection, and ideally incorporate designs that allow this potential confounder to be assessed directly.

## 5. Conclusions

The present study provides preliminary in vivo evidence that 20-gluco-Rf is associated with antidepressant-like and cognition-related behavioral effects in an L-AAA-induced mouse model. These behavioral changes were accompanied by reduced mRNA expression of proinflammatory mediators, including IL-1β, IL-6, TNF-α, and LCN2, as well as changes in GFAP and IBA-1 protein expression in the PFC. Because the present study was limited by a single exploratory dose, modest final sample sizes, absence of a saline-infused surgical control group, lack of direct histological validation, and mRNA-level assessment of inflammatory mediators, further studies are required to clarify the dose–response profile, molecular mechanisms, and therapeutic relevance of 20-gluco-Rf.

## Figures and Tables

**Table 1:** DNA Sequences of the Primers Used for Real-Time PCR.

Genes	Primer Sequences	GenBank Accession Numbers
IL-1β	(F)TGGACCTTCCAGGATGAGGACA (R) GTTCATCTCGGAGCCTGTAGTG	NM_008361
IL-6	(F) AGTTGCCTTCTTGGGACTGA (R) TCCACGATTTCCCAGAGAAC	NM_031168
TNF-α	(F) ATGGCCTCCCTCTCAGTTC (R) TTGGTGGTTTGCTACGACGTG	NM_013693
LCN2	(F) ATGTCACCTCCATCCTGGTC (R) CACACTCACACCCATTCAG	AA087193
GAPDH	(F) TGGGCTACACTGAGCACAG (R) GGGTGTCGCTGTGAAGTCA	NM_008084

## Data Availability

Data supporting the findings of this study are available from the corresponding author upon request.

## Abbreviations

20-gluco-Rf	20-glucoginsenoside Rf
FST	Forced swim test
GFAP	Glial fibrillary acidic protein
IL-1β	Interleukin-1 beta
IL-6	Interleukin-6
IL	Interleukin
L-AAA	L-alpha-aminoadipic acid
LCN2	Lipocalin-2
MDD	Major depressive disorder
NORT	Novel object recognition test
OFT	Open-field test
PFC	Prefrontal cortex
SEM	Standard error of the mean
TNF-α	Tumor necrosis factor alpha
TST	Tail suspension test
Veh	Vehicle
